# Reference values for the short forms of the Singapore Caregiver Quality of Life Scale

**DOI:** 10.1186/s41687-021-00290-5

**Published:** 2021-01-29

**Authors:** Chun Fan Lee, Hwee Lin Wee, Irene Teo, Geok Ling Lee, Julian Thumboo, Yin Bun Cheung, Shirlyn H. S. Neo

**Affiliations:** 1 Centre for Quantitative Medicine, Duke-NUS Medical School, Level 6, Academia, 20 College Road, Singapore, 169856 Singapore; 2 Saw Swee Hock School of Public Health, National University of Singapore (NUS), National University Health System (NUHS), 12 Science Drive 2, Singapore, 117549 Singapore; 3 Department of Pharmacy, Faculty of Science, National University of Singapore, 18 Science Drive 4, Singapore, 117559 Singapore; 4 Division of Supportive and Palliative Care, National Cancer Centre Singapore, 11 Hospital Drive, Singapore, 169610 Singapore; 5 Lien Centre for Palliative Care, Duke-NUS Medical School, 20 College Road, Singapore, 169856 Singapore; 6 Department of Social Work, National University of Singapore, 3 Arts Link, Singapore, 117570 Singapore; 7 Program in Health Services & Systems Research, Duke-NUS Medical School, 20 College Road, Singapore, 169856 Singapore; 8 Department of Rheumatology and Immunology, Singapore General Hospital, Outram road, Singapore, 169608 Singapore; 9 Centre for Child Health Research, Tampere University, Arvo Ylpönkatu 34, 33520 Tampere, Finland

**Keywords:** Caregivers, Neoplasms, Reference values, Quality of life, Singapore caregiver quality of life scale, SCQOLS-15, SCQOLS-10, Surveys and questionnaires

## Abstract

**Purpose:**

The 15- and 10-item short forms of the Singapore Caregiver Quality of Life Scale (SCQOLS-15 and SCQOLS-10) were recently developed as a quick assessment of caregiver quality of life. Reference values describing the distribution of the total and domain scores are available for the full-length version, but they are not yet available for the short forms. This study aimed to estimate the reference values for the short forms.

**Methods:**

Data from a cross-sectional survey of 612 family caregivers of patients with advanced cancer in Singapore were fitted in quantile regression models. Percentiles were estimated by regressing the short forms’ scores on caregiver characteristics. Classification by the reference values for the short forms and the full-length version were compared and agreement was evaluated.

**Results:**

The caregiver’s role in caring for the patient and the patient’s performance status were associated with the percentiles of the total scores and most domain scores (each Bonferroni-adjusted *p*-value, P_B_, < 0.05). Higher-educated caregivers were categorized into higher percentiles according to the SCQOLS-15 and SCQOLS-10 total scores and the SCQOLS-15 Mental Well-being and Financial Well-being domain scores (each P_B_ < 0.05). Ethnicity was associated with the SCQOLS-15 Physical Well-being and Experience & Meaning domains (each P_B_ < 0.05). The percentiles for the short forms showed moderate to substantial agreement with those for the full-length version in terms of classifying caregivers into percentile intervals (quadratic-weighted Kappa = 0.72 to 0.92).

**Conclusion:**

Reference values for the SCQOLS-15 and SCQOLS-10 were estimated in relation to caregiver characteristics to facilitate interpretation of the short form scores.

**Supplementary Information:**

The online version contains supplementary material available at 10.1186/s41687-021-00290-5.

## Introduction

Cancer is a major chronic disease that not only affects patients but also imposes burden and stress on family caregivers, leading to deterioration in caregiver quality of life (QoL). Despite this, there has been a shortage of caregiver QoL measurement scales, especially for Asian populations [1–3]. A qualitative study of family caregivers of patients with advanced cancer in Singapore revealed that existing caregiver QoL instruments developed in the West were inadequate for use in the Asian population [4]. To fill this gap, the Singapore Caregiver Quality of Life Scale (SCQOLS) was developed among family caregivers of cancer patients in Singapore [3].

The SCQOLS is a 51-item questionnaire containing 5 domains [3]. Each domain provides a domain score, and a total score is given by the weighted average of the 5 domain scores. The validity and reliability of the SCQOLS have been evaluated. The questionnaire is available in English and Chinese versions and has been shown to give equivalent mean scores after controlling for demographic variables [5, 6]. Two short forms have also been assessed for their validity and reliability, namely the SCQOLS-15 and the SCQOLS-10 [7]. They contain 15 and 10 items, respectively. The SCQOLS-15 retains the 5-domain structure and can be used to assess domain-specific and overall levels of QoL, whereas the SCQOLS-10 is used to assess the overall level of QoL only. The questionnaires are available to the public (https://www.duke-nus.edu.sg/lcpc/resources/scqols-request-forms).

Although the total and domain scores are scaled to range from 0 to 100, they do not provide a ratio level of measurement. To facilitate interpretation, reference values for the SCQOLS were constructed to indicate the distribution of the total and domain scores [5]. Users of the SCQOLS can then check whether the domain and total scores indicate a high QoL level, indicate a low QoL level, or fall outside the typical range of the population. The reference values were produced by estimating various percentiles in relation to caregivers’ and patients’ characteristics using quantile regression. In the conventional approach of developing reference values, percentiles are separately estimated for subgroups with different characteristics such as age. To maintain acceptable precision, each subgroup requires a sufficiently large sample size (≥ 200); hence, this approach requires a large total sample size [8]. In contrast, quantile regression pools all observations in the modeling, and utilizes information from the whole sample rather than the specific subgroup, thus reducing the sample size requirement [9, 10].

Reference values for the short forms of the SCQOLS have not been developed. The primary objective of this study was to estimate quantile regression models that regress the SCQOLS-15 and SCQOLS-10 scores on caregiver and patient characteristics. These models can then generate percentiles that serve as reference values for different characteristic-specific subgroups of family caregivers of cancer patients. Our secondary objective was to compare the performance of the quantile regression and conventional approach in the construction of reference values in QoL research, using the SCQOLS-15 total score as an illustration

## Methods

### Design and setting

This is a secondary analysis of a study that developed the SCQOLS by interviewing 612 family caregivers [3]. These caregivers were recruited in 2016 to 2017 from the Nation Cancer Centre, Singapore and the Singapore General Hospital, the largest public providers of outpatient and inpatient cancer cares in Singapore [11]. The study was approved by the Singapore Health Services Centralized Institutional Review Board (#2016/2243). Written informed consent was given by each participant.

Details of the study design have been described in previous reports [3, 5, 7]. Briefly, family caregivers of patients with stage III or IV solid tumors who were receiving care from the two abovementioned institutions were invited to participate. A family caregiver is defined as a family member who directly provides or ensures the supply of care to meet the patient’s day-to-day and healthcare needs, or makes decisions on how these needs are met. Caregivers who were 21 years of age or older, able to communicate in either English or Chinese (Mandarin), aware of the patient’s diagnosis, and not in the bereavement stage were eligible. For each patient, only one caregiver was recruited; if two or more caregivers of the same patient were eligible and agreed to participate, we recruited the one who was most involved in taking care of the patient.

The study consisted of a baseline and a follow-up survey. In the present report, data were extracted from the baseline survey. Each participant was invited to answer a questionnaire package in either English or Chinese according to their preference. Caregivers were asked to self-administer the questionnaires, but interviewer-administration was allowed upon request.

### Questionnaire and measurements

The questionnaire package included the SCQOLS and some questions on caregiver demographics, caregiving background and patient health characteristics. The 51-item SCQOLS consists of 5 domains: Physical Well-being (PW; 12 items), Mental Well-being (MW; 10 items), Experience & Meaning (EM; 12 items), Impact on Daily Life (DL; 13 items) and Financial Well-being (FW; 4 items) [3]. Each item is rated on a 5-point scale, from not at all (0) to very much (4). For each domain, a domain score is calculated as the mean score of the items in that domain after recoding negatively worded items such that a higher score indicates a better QoL. The mean score was then multiplied by 25 to rescale it to the 0 to 100 scale. Missing values are imputed by the half-rule [12]. The QoL total score is a weighted average of the 5 domain scores, with the weights being the number of items in the 5 domains. The SCQOLS-15 has the same 5-domain structure [7]. Each domain of the SCQOLS-15 is constituted by 2 to 4 items from the respective domain of the SCQOLS. The SCQOLS-15 total and domain scores are calculated in the same manner as the SCQOLS scores. The SCQOLS-10 retains 2 items from each of the 5 domains of the SCQOLS, from which a total score is computed using the above algorithm. The weights for calculating the total score for the SCQOLS-15 and SCQOLS-10 are the numbers of items in the domains of the full-length SCQOLS, not those of the short forms [7]. In this study, the full-length SCQOLS was administered, and the short form scores were calculated by extracting the respective items for the SCQOLS-15 and SCQOLS-10.

The caregivers were asked to indicate his/her role in carrying out caregiving duties for the patient by selecting one of the following: “the only person” (coded as 0), “the primary person” (coded as 1) or “one of the few persons” (coded as 2). Hereafter this variable is referred to as “caregiver role”. Caregivers with a higher coded value in caregiver role were expected to have a better QoL. The caregivers were also asked to specify the patient’s cancer diagnosis and to rate the patient’s performance status. The performance status score is strongly correlated with cancer patient QoL and ranges from 0 (without symptoms) to 4 (bedridden), excluding a score of 5 (death) which is not applicable in the study [13–15].

### Statistical analysis

Quantile regression was applied to evaluate how the 10th, 25th, 50th, 75th and 90th percentiles of the SCQOLS-15 total and domain scores and the SCQOLS-10 total score were associated with caregiver and patient characteristics. While a least square regression minimizes the sum of the squared deviations to estimate the mean of the response variable, an unweighted quantile regression (also known as median regression) minimizes the sum of the absolute deviations to estimate the median (50th percentile) [10, 16]. By allocating appropriate weights to the deviations, the corresponding percentiles are estimated. For example, for estimating the 10th percentile, a weight of 0.9 is assigned to the negative deviations while a weight of 0.1 is assigned to the positive deviations. An advantage of quantile regression is that it does not require any distributional assumptions such as a normal distribution or homoskedasticity of the error terms. For parsimony, the final regression equations do not include the interaction effect between different predictors. The predictor variables considered here were caregiver demographics including age, sex, ethnicity (Chinese, Malay, Indian or Others), education level (tertiary, secondary and primary or below), caregiver role, patient performance status and cancer diagnosis, mode of survey administration and the language version used.

For each total or domain score, an initial model that included all predictor variables was first fitted. Backward model selection with Bonferroni adjustment for multiplicity was employed. Since 5 percentiles were fitted in each model, a predictor variable was kept in the next model if its regression coefficient showed a Bonferroni-adjusted *p*-value < 0.05, or equivalently a *p-*value < 0.01 in at least one of the 5 percentile equations; otherwise the predictor variable was removed. To examine the linearity of age, we fitted an additional model with a quadratic term for age (age × age). An insignificant coefficient indicated the lack of a turning point in the relationship between age and the percentile. We compared two different models, one treating caregiver role and performance status (both ordinal level variables) as categorical predictors, and the other treating them as continuous predictors. For each model, the absolute deviations between the observed score and the predicted median were computed. If the mean absolute deviations of the two models had a small, insignificant difference when examined by a paired t-test, the simpler model that treats them as continuous predictors was chosen for parsimony. For other categorical predictors with more than two levels, if one or more levels obtained a *p*-value < 0.01, the predictor was retained in the next model, but levels would be combined if the difference between them were not statistically significant. After obtaining the final models, we added 2-way interaction terms between the significant predictors in each model to examine the interaction effects. A resampling method with 1000 replicates was used to estimate the standard errors and *p*-values.

The agreement of the reference values between the full-length version and the two short form versions was also assessed. We first divided the caregivers into different subgroups based on their characteristics, and then for each subgroup, we calculated the reference values according to the final model for the short forms’ scores. The reference values for the full-length version of the SCQOLS were also calculated for each subgroup [5]. We classified the caregivers into one of the six percentile intervals by their observed scores: (A) < 10th percentile, (B) 10th to < 25th percentile, (C) 25th to < 50th percentile, (D) 50th to < 75th percentile, (E) 75th to < 90th percentile, and (F) ≥ 90th percentile. Unweighted and quadratic-weighted Kappa statistics were used to evaluate the agreement between the short-form and full-length classifications [17]. All statistical analyses were performed in SAS version 9.4 (SAS Institute, Cary, NC, USA).

## Results

### Descriptive summary

The characteristics of the 612 caregivers have been reported previously [3, 5, 7]. In brief, the sample mean age was 48 (standard deviation = 14, ranging from 21 to 79) years; 61.0% were female; 85.1% were ethnic Chinese; 15.2% had received a primary education or below; 49.7% responded to the English questionnaire package; 90.0% self-administered the questionnaire. In total, 20.6%, 35.5% and 44.0% of the caregivers were the only person, the primary person and one of the few persons in the family who took care of the patient, respectively. The patients’ performance status ranged from 0 (11.6%) to 4 (13.9%), with a mode of 1 (33.5%). Colorectal (23.7%), lung (21.1%) and breast (11.8%) cancer were the major cancer types. Other diagnoses included liver (6.0%), prostate (5.6%), pancreas (4.6%), stomach (4.4%), ovary (3.6%), kidney (2.6%), esophageal (1.8%), nose (1.8%), and bile duct (1.6%) cancer; a variety of other rare diagnoses were also reported (< 1% each). These less-common diagnoses were combined as “Others”.

### 15-item version total score

Table 1 summarizes the results of the initial quantile regression model for the SCQOLS-15 QoL total score. Sex, ethnicity and mode of survey administration were not significantly associated with any of the percentiles (each *p*-value > 0.01). After removing these predictors in the next model (details not shown), age, survey language version and patient diagnosis became insignificant. For education level, the coefficients were not significantly different between the tertiary and secondary levels, so they were combined into one level. In the additional model examining the linearity of age, the coefficients of the quadratic term for age were all insignificant, implying that the percentiles for the SCQOLS-15 QoL total score increased with age. The coefficients for caregiver role showed a monotonic increasing trend in all 5 percentiles. The coefficients for performance status showed a monotonic decreasing trend with the exception that, comparing the coefficients for the association of performance status 3 and 4 with the 75th percentile, there was an insignificant increase from − 7.2 to − 7.1 (*p*-value = 0.979). The mean absolute deviation of the initial model in which caregiver role and performance status were kept as categorical predictors was 11.36, while that of the model treating them as continuous predictors was 11.38 (*p-*value = 0.599). Therefore, these two predictors were used as continuous predictors in the subsequent analyses. Education level, caregiver role and performance status were retained in the final model (Table 2). To facilitate application of the findings, we developed an Excel spreadsheet to compute the percentiles by specifying the respondent’s and patient’s characteristics (Online Supplementary Material).

**Table 1:** Percentiles of the SCQOLS-15 QoL total score, initial model

Predictor	10th percentile		25th percentile		50th percentile		75th percentile		90th percentile	
	Coef	95% CI	Coef	95% CI	Coef	95% CI	Coef	95% CI	Coef	95% CI
Age per 10 years	−0.3	(−2.5, 1.9)	0.6	(−2.5)	0.5	(−0.8, 1.7)	1.4	(0.3, 2.5)	1.7*	(0.4, 3.0)
Sex										
Female	0		0		0		0		0	
Male	2.7	(−2.2, 7.7)	−0.5	(−4.4, 3.3)	0.5	(−2.2, 3.2)	0.0	(−2.2, 2.2)	−1.6	(−4.3, 1.1)
Ethnicity										
Chinese	0		0		0		0		0	
Malay	8.5	(−1.6, 18.6)	5.1	(−2.3, 12.5)	4.6	(−0.8, 10.0)	6.1	(1.0, 11.1)	5.5	(−0.1, 11.1)
Indian	8.1	(−19.5, 35.6)	4.5	(−8.8, 17.8)	−0.9	(−8.2, 6.5)	0.8	(−7.3, 9.0)	−3.1	(−14.0, 11.5)
Others	8.3	(16.8, 33.4)	6.5	(−4.5, 17.5)	2.6	(−5.6, 10.7)	0.9	(−9.1, 11.0)	1.0	(−11.9, 13.9)
Education level										
Tertiary	0		0		0		0		0	
Secondary	−4.3	(−9.6, 1.0)	−0.8	(−5.8, 4.3)	1.7	(−1.4, 4.9)	−0.8	(−3.6, 2.1)	0.1	(−3.2, 3.5)
Primary/below	−1.3	(−9.7, 7.2)	−6.8	(−14.8, 1.3)	−2.8	(−7.3, 1.8)	−6.0*	(−10.1, −1.9)	−7.4*	(−12.5, −2.3)
Language										
English	0		0		0		0		0	
Chinese	7.5*	(2.4, 12.6)	1.1	(−3.7, 6.0)	2.1	(−0.9, 5.2)	1.5	(−1.2, 4.2)	2.1	(−1.2, 5.3)
Mode of survey administration										
Coef = 0 for each percentile										
Interviewer	−7.4	(−20.2, 5.4)	3.0	(−6.9, 13.0)	−4.5	(−9.6, 0.5)	−3.0	(−8.9, 2.9)	−2.1	(−7.6, 3.4)
Caregiver role										
0	0		0		0		0		0	
1	5.3	(−0.9, 11.6)	7.5	(1.7, 13.3)	5.5*	(1.4, 9.6)	3.6	(0.1, 7.2)	1.6	(−2.3, 5.4)
2	14.2*	(8.4, 20.1)	13.5*	(8.3, 18.8)	9.4*	(5.5, 13.4)	5.6*	(1.8, 9.5)	4.3	(0.5, 8.0)
Patient’s performance status										
0 (Best)	0		0		0		0		0	
1	−0.8	(−9.4, 7.8)	−7.0	(−13.3, −0.6)	−4.3	(−8.3, −0.2)	−3.0	(−6.7, 0.8)	−2.0	(−6.8, 2.8)
2	−8.3	(−20.1, 3.6)	−12.1*	(−20.1, −4.2)	−7.6	(−13.8, −1.4)	−4.8	(−9.4, −0.1)	−2.5	(−8.1, 3.1)
3	−11.1	(−19.7, −2.5)	−15.3*	(−22.1, −8.5)	−9.7	(−14.3, −5.2)	−7.2*	(−11.0, −3.3)	−4.3	(−9.5, 0.9)
4 (Worst)	−20.2*	(−29.8, −10.5)	−21.1*	(−29.6, −12.6)	−15.4	(−22.5, −8.3)	−7.1*	(−12.0, −2.2)	−7.6	(−13.7, −1.6)
Patient’s Diagnosis										
Colorectal cancer	0		0		0		0		0	
Breast cancer	6.8	(−3.5, 17.2)	4.7	(−2.9, 12.2)	4.2	(−0.8, 9.3)	−1.8	(−7.3, 3.7)	3.5	(−2.0, 8.9)
Lung cancer	8.5	(1.9, 15.1)	2.3	(−4.3, 8.9)	1.3	(−2.9, 5.5)	−0.2	(−3.8, 3.4)	−1.4	(−5.1, 2.4)
Others	8.2*	(2.1, 14.4)	3.8	(−1.4, 9.0)	1.2	(−2.2, 4.6)	−1.8	(−4.8, 1.1)	−1.4	(−5.0, 2.2)
Intercept	39.0*	(25.1, 52.9)	57.8*	(45.7, 69.9)	68.2*	(60.0, 76.4)	75.2*	(67.0, 83.4)	79.8*	(71.2, 88.4)

*P-value < 0.01

Abbreviations: *Coef* coefficient, *CI* confidence interval

**Table 2:** Percentiles of the SCQOLS-15 QoL total score, final model

Predictor	10th percentile		25th percentile		50th percentile		75th percentile		90th percentile	
	Coef	95% CI	Coef	95% CI	Coef	95% CI	Coef	95% CI	Coef	95% CI
Education level										
Secondary/above	0		0		0		0		0	
Primary/below	−4.1	(−10.3, 2.2)	−5.9	(−13.3, 1.6)	−3.9	(−8.2, 0.3)	−4.2*	(−6.9, −1.5)	−5.1	(−10.0, −0.1)
Caregiver role	6.9*	(3.7, 10.0)	6.0*	(3.5, 8.6)	3.5*	(1.7, 5.3)	1.2	(−0.6, 3.1)	1.0	(−0.8, 2.8)
Performance status	−5.6*	(−7.7, −3.4)	−4.6*	(−6.4, −2.7)	−3.5*	(−4.8, −2.1)	−2.0*	(−2.8, −1.3)	−2.1*	(−3.3, −1.0)
Intercept	52.5*	(45.7, 59.2)	63.6*	(58.6, 68.5)	75.2*	(71.7, 78.7)	83.7*	(80.7, 86.6)	90.5*	(87.0, 94.0)

*P-value < 0.01

Abbreviations: *Coef* coefficient, *CI* confidence interval

Example: The 10th percentile of a caregiver with primary education who is the primary person (caregiver role = 1) giving care to a patient who has no symptoms (performance status = 0) is 52.5 + (− 4.1) + (1 × 6.9) + (0 × [−5.6]) = 55.3

### 15-item version domain scores

The final models for the 5 SCQOLS-15 domain scores are presented in Table 3. Similar to the modeling of QoL total score, caregiver role and performance status were significant predictors of all domain scores except Experience & Meaning. There were heavy ceiling effects in the Physical Well-being (38.6%), Impact on Daily Life (42.3%) and Financial Well-being (31.0%) scores. Hence the quantile regression equation for the 75th and 90th percentiles of these scores may not be estimable. In this case, the intercept was set as 100 and the regression coefficients for the predictors were all zero.

**Table 3:** Percentiles of domain scores of the SCQOLS-15, final model

Predictor	10th percentile		25th percentile		50th percentile		75th percentile		90th percentile	
	Coef	95% CI	Coef	95% CI	Coef	95% CI	Coef	95% CI	Coef	95% CI
*Physical Well-being*										
Ethnicity										
Chinese	0		0		0		0		0	
Indian	−16.7	(−73.7, 40.4)	0.0	(−26.7, 26.7)	0.0	(−13.3, 13.3)	0	(0, 0)	0	(0, 0)
Malay and Others	16.7	(2.0, 31.4)	8.3*	(2.4, 14.3)	4.2	(−0.2, 8.6)	0	(0, 0)	0	(0, 0)
Caregiver role	16.7*	(12.4, 20.9)	8.3*	(2.9, 13.8)	6.4*	(3.7, 8.8)	0	(0, 0)	0	(0, 0)
Performance status	−4.2*	(−7.3, −1.1)	−8.3*	(−10.9, −5.8)	−4.2*	(−5.5, −2.8)	0	(0, 0)	0	(0, 0)
Intercept	37.5*	(27.0, 48.0)	65.8*	(65.8, 84.2)	87.5*	(82.4, 92.6)	100	(100, 100)	100	(100, 100)
*Mental Well-being*										
Education level										
Secondary/above	0		0		0		0		0	
Primary/below	−12.5*	(−18.7, −6.3)	−12.5*	(−21.2, −3.8)	−6.3	(−14.7, 2.2)	5.0	(−8.9, 18.9)	4.2	(−6.2, 14.5)
Caregiver role	2.1	(−1.7, 5.9)	4.2	(0.9, 7.4)	6.3*	(2.0, 10.5)	5.0	(0.9, 9.1)	4.2	(0.1, 8.2)
Performance status	−4.2*	(−6.5, −1.9)	−4.2*	(−6.0, −2.3)	−6.2*	(−8.3, −4.2)	−3.3	(−5.9, −0.8)	−4.2*	(−7.1, −1.3)
Intercept	25.0*	(15.7, 34.3)	37.5*	(30.7, 44.3)	52.1*	(44.9, 59.3)	66.7*	(59.4, 73.9)	83.3*	(75.6, 91.1)
*Experience & Meaning*										
Ethnicity										
Chinese	0		0		0		0		0	
Others	12.5	(−2.0, 27.0)	12.5*	(5.5, 19.5)	18.8*	(12.2, 25.3)	12.5*	(7.8, 17.2)	6.3*	(4.4, 8.1)
Intercept	31.3*	(27.0, 35.5)	50.0*	(44.7, 55.3)	62.5*	(56.5, 68.5)	81.3*	(79.7, 82.8)	93.8*	(91.9, 95.6)
*Impact on Daily Life*										
Caregiver role	12.5*	(5.5, 19.5)	8.3*	(4.5, 12.1)	4.2*	(1.8, 6.6)	0	(0, 0)	0	(0, 0)
Performance status	−12.5*	(−16.9, −8.1)	−8.3*	(−11.0, −5.7)	−4.2*	(−5.0, −3.3)	0	(0, 0)	0	(0, 0)
Intercept	54.2*	(38.7, 69.6)	75.0*	(68.6, 81.4)	91.7*	(87.7, 95.7)	100	(100, 100)	100	(100, 100)
*Financial Well-being*										
Age per 10years	0.0	(−5.8, 5.8)	1.8	(−1.9, 5.6)	4.6*	(1.8, 7.5)	0.0	(−1.9, 1.9)	0	(0, 0)
Education level										
Secondary/above	0		0		0		0		0	
Primary/below	−13.0	(−29.0, 3.7)	−17.0	(−31.0, −3.3)	−21.0*	(−33.0, −8.9)	−13.0	(−22.0, −2.9)	0	(0, 0)
Caregiver role	12.5	(3.7, 21.3)	17.2*	(12.0, 22.4)	13.9*	(8.9, 18.9)	0.0	(−4.2, 4.2)	0	(0, 0)
Performance status	−4.2	(−9.2, 0.9)	−5.6*	(−8.9, −2.3)	−1.9	(−4.6, 0.9)	0.0	(−0.9, 0.9)	0	(0, 0)
Intercept	16.7	(−21.0, 54.3)	34.1*	(12.6, 55.5)	42.6*	(23.8, 61.3)	100.0	(84.3, 100.0)	100	(100, 100)

*P-value <0.01

Abbreviations: *Coef* coefficient, *CI* confidence interval

Example: The 10th percentile of the domain scores of a 50-year old, ethnic Malay caregiver with primary education who is the primary person (caregiver role = 1) giving care to a patient who has no symptoms (performance status = 0) are:

Physical Well-being: 37.5 + 16.7 + (1 × 16.7) + (0 × [−4.2]) = 70.9

Mental Well-being: 25.0 + (−12.5) + (1 × 2.1) + (0 × [−4.2]) = 14.6

Experience & Meaning: 31.3 + 12.5 = 43.8

Impact on Daily Life: 54.2 + (1 × 12.5 + (0 × [−12.5]) = 66.7

Financial Well-being: 16.7 + (5 × 0.0) + (−13.0) + (1 × 12.5) + (0 × [−4.2]) = 16.2

For the Physical Well-being domain, ethnicity was significantly associated with the percentiles. Compared with ethnic Chinese caregivers, Malay and Others showed a higher 10th percentile, but Indian showed a lower 10th percentile. Hence, ethnicity was regrouped by combining Malay and Others as one level. Education level was not associated with any of the percentiles.

For the Mental Well-being domain, primary education or below was associated with lower 10th and 25th percentiles. There was no significant difference between the tertiary and secondary levels (each *p*-value > 0.05); hence, they were combined.

Only ethnicity was related to the Experience & Meaning domain score. The three non-Chinese groups were not significantly different from each other (each pairwise *p-*value > 0.1) but showed higher scores than the ethnic Chinese group. Therefore, these three groups were regrouped as one level.

The Impact on Daily Life domain score was only significantly associated with caregiver role and patient performance status.

Age and education level were significantly related to the median of the Financial Well-being domain score. There was no significant difference between the tertiary and secondary education levels (each *p*-value > 0.05); hence, they were combined.

### 10-item version total score

The final model for the SCQOLS-10 QoL total score is presented in Table 4. Similar to the SCQOLS-15, the SCQOLS-10 QoL total score was associated with education level, caregiver role and performance status. For education level, there was no significant difference in the coefficients for tertiary and secondary levels, so they were grouped as one level.

**Table 4:** Percentiles of the SCQOLS-10 QoL total score, final model

Predictor	10th percentile		25th percentile		50th percentile		75th percentile		90th percentile	
	Coef	95% CI	Coef	95% CI	Coef	95% CI	Coef	95% CI	Coef	95% CI
Education level										
Secondary/above	0		0		0		0		0	
Primary/below	−2.1	(−11.7, 7.6)	−5.8	(−11.1, −0.5)	−6.6*	(−10.9, −2.3)	−5.9*	(−8.8, −3.1)	−5.4	−12.0, 1.2)
Caregiver role	8.5*	(4.9, 12.2)	6.3*	(3.8, 8.7)	3.3*	(1.4, 5.2)	2.6*	(0.9, 4.3)	1.5	(−0.4, 3.3)
Performance status	−5.3*	(−7.5, −3.1)	−4.6*	(−6.0, −3.1)	−3.8*	(−5.1, −2.6)	−1.9*	(−2.8, −1.0)	−1.7*	(−2.8, −0.6)
Intercept	48.7*	(40.6, 56.9)	63.9*	(59.3, 68.5)	77.1*	(73.7, 80.5)	82.6*	(78.6, 86.5)	90.9*	(87.7, 94.1)

*P-value <0.01

Example: The 10th percentile of a caregiver with primary education who is the primary person (caregiver role = 1) giving care to a patient who has no symptoms (performance status = 0) is 48.7 + (−2.1) + (1 × 8.5) + (0 × [−5.3]) = 55.1

### Examination of interaction effects

Out of 105 tests of interaction, statistically significant interaction effects were only found on two occasions: between caregiver role and patient performance status for the 50th percentile of the Physical Well-being (*p*-value = 0.004) and Impact to Daily Life (*p-*value < 0.001) domain scores. Since the point estimates for these interaction terms were small, adding them to the models made a small, practically negligible difference from the reference values. Therefore, these terms were not included in the final models for parsimony.

### Agreement between full-length and short versions

Table 5 cross tabulates the frequencies of caregivers falling into each percentile interval of the full-length version and the short forms. For example, consider two caregivers with the same SCQOLS-15 QoL total score of 82. One belongs to the subgroup of caregivers with secondary or above education who were the only persons giving care to a patient with performance status 0. The 50th and 75th percentiles of the SCQOLS-15 QoL total score for this subgroup were 75.2 and 83.7, respectively, according to Table 2. This caregiver falls into the interval (D) 50th to < 75th percentile. The other caregiver belongs to the subgroup of caregivers with secondary or above education who were the only persons giving care to a patient with performance status 1. The 75th and 90th percentiles were 81.7 and 88.4, respectively. Therefore, this caregiver falls into the interval (E) 75th to < 90th percentile. Similarly, caregivers were also classified according to their SCQOLS QoL total score and the previously published full-length version SCQOLS reference values [5]. Kappa statistics between the full-length version and the short forms are also presented. Three hundred eighty-eight caregivers (63.4%) were classified into the same interval according to the SCQOLS-15 and SCQOLS QoL total scores (diagonal entries of the first panel), and 214 (35.0%) were classified into the adjacent interval. The quadratic-weighted Kappa statistic was 0.90 (0.88, 0.92). A similar classification comparison was also performed for the SCQOLS-15 domain scores and the SCQOLS-10 total score with the full-length version. Since the 75th percentile for the SCQOLS-15 Physical Well-being and Impact on Daily Life scores was 100, the intervals (E) 75th to < 90th percentile and (F) ≥ 90th percentile were combined. The quadratic-weighted Kappa statistic for the domain scores between the SCQOLS and SCQOLS-15 ranged between 0.72 and 0.92. The quadratic-weighted Kappa statistic between the SCQOLS and SCQOLS-10 QoL total scores was 0.87 (0.85, 0.89), with 363 (59.3%) caregivers classified into the same interval by the two scores.

**Table 5:** Agreement of reference values between the full-length version and short forms

*SCQOLS QoL total score*	*SCQOLS-15 QoL total score*						
	(A)	(B)	(C)	(D)	(E)	(F)	Total
(A) < 10th percentile	50	12	2	0	0	0	64
(B) 10th to < 25th percentile	9	60	18	0	0	0	87
(C) 25th to < 50th percentile	0	20	100	31	2	0	153
(D) 50th to < 75th percentile	0	1	29	93	26	1	150
(E) 75th to < 90th percentile	0	0	1	28	45	22	96
(F) ≥ 90th percentile	0	0	0	3	19	40	62
Total	59	93	150	155	92	63	612
Kappa statistic: Unweighted = 0.55 (0.50, 0.60), Quadratic-weighted = 0.90 (0.88, 0.92)							
*SCQOLS Physical Well-being score*	*SCQOLS-15 Physical Well-being score*						
	(A)	(B)	(C)	(D)	(E) & (F)^a^		Total
(A) < 10th percentile	39	15	3	0	0		57
(B) 10th to < 25th percentile	6	44	45	1	2		98
(C) 25th to < 50th percentile	0	11	72	27	25		135
(D) 50th to < 75th percentile	1	1	39	51	102		194
(E) 75th to < 90th percentile	0	0	1	17	36		54
(F) ≥ 90th percentile	0	0	0	3	71		74
Total	46	71	160	99	236		612
Kappa statistic: Unweighted = 0.33 (0.29, 0.38), Quadratic-weighted = 0.72 (0.68, 0.76)							
*SCQOLS Mental Well-being score*	*SCQOLS-15 Mental Well-being score*						
	(A)	(B)	(C)	(D)	(E)	(F)	Total
(A) < 10th percentile	34	22	2	0	0	0	58
(B) 10th to < 25th percentile	18	36	23	2	0	0	79
(C) 25th to < 50th percentile	1	33	87	38	5	0	164
(D) 50th to < 75th percentile	0	1	36	93	26	3	159
(E) 75th to < 90th percentile	0	0	0	28	40	24	92
(F) ≥ 90th percentile	0	0	0	1	10	49	60
Total	53	92	148	162	81	76	612
Kappa statistic: Unweighted = 0.45 (0.40, 0.50), Quadratic-weighted = 0.87 (0.85, 0.89)							
*SCQOLS Experience & Meaning score*	*SCQOLS-15 Experience & Meaning score*						
	(A)	(B)	(C)	(D)	(E)	(F)	Total
(A) < 10th percentile	41	16	0	0	0	0	57
(B) 10th to < 25th percentile	8	52	23	4	1	0	88
(C) 25th to < 50th percentile	3	24	61	58	9	0	155
(D) 50th to < 75th percentile	0	0	19	86	27	10	142
(E) 75th to < 90th percentile	0	0	4	26	42	29	101
(F) ≥ 90th percentile	0	0	0	6	13	50	69
Total	52	92	107	180	92	89	612
Kappa statistic: Unweighted = 0.44 (0.39, 0.49), Quadratic-weighted = 0.85 (0.83, 0.87)							
*SCQOLS Impact on Daily Life score*	*SCQOLS-15 Impact on Daily Life score*						
	(A)	(B)	(C)	(D)	(E) & (F)^a^		Total
(A) < 10th percentile	43	16	2	0	0		61
(B) 10th to < 25th percentile	13	38	28	6	3		88
(C) 25th to < 50th percentile	0	20	78	28	31		157
(D) 50th to < 75th percentile	0	1	24	39	88		152
(E) 75th to < 90th percentile	0	0	4	13	72		89
(F) ≥ 90th percentile	0	0	0	0	65		65
Total	56	75	136	86	259		612
Kappa statistic: Unweighted = 0.32 (0.27, 0.36), Quadratic-weighted = 0.73 (0.69, 0.76)							
*SCQOLS Financial Well-being score*	*SCQOLS-15 Financial Well-being score*						
	(A)	(B)	(C)	(D)	(E)	(F)	Total
(A) < 10th percentile	45	20	0	0	0	0	65
(B) 10th to < 25th percentile	7	62	24	2	0	1	96
(C) 25th to < 50th percentile	0	15	105	16	0	5	141
(D) 50th to < 75th percentile	0	0	23	81	5	22	131
(E) 75th to < 90th percentile	0	0	0	13	4	11	28
(F) ≥ 90th percentile	0	0	0	0	0	151	151
Total	52	97	152	112	9	190	612
Kappa statistic: Unweighted = 0.66 (0.62, 0.71), Quadratic-weighted = 0.92 (0.90, 0.94)							
*SCQOLS QoL total score*	*SCQOLS-10 QoL total score*						
	(A)	(B)	(C)	(D)	(E)	(F)	Total
(A) < 10th percentile	47	15	1	1	0	0	64
(B) 10th to < 25th percentile	11	51	24	1	0	0	87
(C) 25th to < 50th percentile	1	21	97	27	7	0	153
(D) 50th to < 75th percentile	0	4	29	84	30	3	150
(E) 75th to < 90th percentile	0	0	3	35	41	17	96
(F) ≥ 90th percentile	0	0	0	5	14	43	62
Total	59	91	154	153	92	63	612
Kappa statistic: Unweighted = 0.50 (0.45, 0.55), Quadratic weighted = 0.87 (0.85, 0.89)							

^a^The 75th percentile for the SCQOLS-15 Physical Well-being and Impact on Daily Life scores was 100, the intervals (E) 75th to < 90th percentile and (F) ≥ 90th percentile were combined

### Comparison between quantile regression and the conventional approach

To compare the performance between quantile regression and the conventional approach of developing reference percentiles for a subgroup using only the observations from the subgroup, we present the predicted percentiles of the SCQOLS-15 QoL total score for four subgroups of caregivers as an illustration. The first two subgroups were the largest in the study sample: caregivers with secondary or above education who were the primary person (subgroup (a), *N* = 86) or one of the few persons (subgroup (b), *N* = 66) giving care to a patient whose performance status was 1 (Table 6). The third and fourth subgroups were those who had a primary or below education, and were the only person (subgroup (c), *N* = 5) or one of the few persons (subgroup (d), no such caregiver in the study sample) giving care to a patient with performance status 2. The SCQOLS-15 QoL total score percentiles and the corresponding standard errors were estimated by the quantile regression model presented in Table 2 as well as by the conventional approach. The point estimates for subgroups (a) and (b) were very similar between the two methods, with a maximum difference of 2.2. On the other hand, the standard errors estimated by quantile regression were consistently smaller than those estimated by the conventional approach. For subgroup (c), the point estimates were different between the two methods, especially for the 25th percentile, and the conventional approach obtained an estimate of 71.2 with a standard error of 17.2. The 5 percentiles estimated by the conventional approach were the 5 observed scores in this subgroup. For subgroup (d), no percentile was estimable by the conventional approach since there were no observations, while quantile regression could still provide estimates using information from other subgroups.

**Table 6:** Predicted percentiles of the SCQOLS-15 QoL total score for selected subgroups of caregivers estimated by quantile regression and the conventional approach

Characteristics	N	Estimation method	10th percentile Estimate (SE)	25th percentile Estimate (SE)	50th percentile Estimate (SE)	75th percentile Estimate (SE)	90th percentile Estimate (SE)
(a) Secondary or above education, one of the few persons giving care to a patient with performance status 1	86	Regression	60.6 (1.8)	71.1 (1.3)	78.8 (1.1)	84.1 (1.1)	90.4 (1.1)
		Conventional	60.5 (3.8)	69.8 (2.3)	76.6 (1.5)	82.2 (1.6)	90.4 (2.4)
(b) Secondary or above education, the primary person giving care to a patient with performance status 1	66	Regression	53.8 (1.8)	65.0 (1.3)	75.2 (1.0)	82.2 (0.8)	89.4 (1.0)
		Conventional	52.9 (5.2)	65.0 (3.5)	75.8 (1.8)	82.7 (2.2)	89.1 (1.7)
(c) Primary or below education, the only person giving care to a patient with performance status 2	5	Regression	37.3 (2.9)	48.5 (3.3)	64.3 (2.4)	75.4 (1.5)	81.2 (2.5)
		Conventional	35.8 (9.6)	71.2 (17.2)	71.2 (14.0)	73.2 (1.5)	73.5 (0.6)
(d) Primary or below education, one of the few persons giving care to a patient with performance status 2	0	Regression	51.0 (3.2)	60.6 (3.9)	71.4 (2.1)	77.9 (1.6)	83.2 (2.6)
		Conventional	Not estimable	Not estimable	Not estimable	Not estimable	Not estimable

Abbreviation: *SE* standard error

## Discussion

The participants’ demographic profile, in terms education, age and sex, was similar to the caregiver profiles shown in other surveys of caregivers [17] and caregivers of cancer patients in Singapore [18]. As such, we believe the sample was representative of the target population.

The SCQOLS is a valuable measure in evaluating Asian family caregivers’ QoL [3]. The abbreviated versions of the SCQOLS-15 and SCQOLS-10 have been demonstrated as alternatives to the full-length version with satisfactory validity and reliability and can serve as a quick assessment of caregiver QoL [5]. In this study, we estimated the reference values for the short forms, which agreed with the set of reference values of the full-length version from two perspectives. The Kappa statistics comparing the proportion of caregivers classified into different percentile intervals for various scores showed moderate to substantial agreement between the short forms and the full-length version [19]. Moreover, the predictor variables retained in the final model for the short forms’ total and domain scores were the same as or a subset of those for the SCQOLS, while the coefficients were also comparable. The results indicated that the interpretations of the reference values were consistent across the full-length and abbreviated versions.

The Physical Well-being, Impact on Daily Life and Financial Well-being domain scores of the SCQOLS-15 presented a heavy ceiling effect, with more than a quarter of the study sample attaining the maximum score of 100. This caused the 75th and/or 90th percentiles to be fixed at 100 but not dependent on the predictor variables. Nevertheless, in clinical practice individuals at the lower end rather than the upper end of the distribution are of greater concern. Caregivers with worse QoL, similar to patients in poorer conditions, usually have a larger need for healthcare support than their healthier counterparts. Therefore, it is essential to distinguish those at the lower end by the 10th and 25th percentiles. In this sample, as only a small proportion of caregivers obtained the minimum of 0 in the total and domain scores, the 10th, 25th and 50th percentiles were estimable and meaningfully associated with caregiver and patient characteristics. Hence, the reference values defined in this study are still useful.

We compared the performance of percentiles estimated by quantile regression and the conventional approach using the SCQOLS-15 total score as an illustration. For reasonably large subgroups of participants such as subgroups (a) and (b) in the illustration, both methods provided valid and similar point estimates, yet the standard errors were consistently smaller in quantile regression than in the conventional approach. When the sample size was small (subgroup (c)), the two methods obtained quite different point estimates, and the standard errors were much larger in the conventional approach than in quantile regression. This suggested that the estimates from the conventional approach were less certain. In the extreme case where there were zero observations (subgroup (d)) in this specific sample, the conventional approach was inapplicable; in contrast, quantile regression was able to use information from other subgroups in the sample to obtain point estimates with reasonable precision. This may facilitate the application when such a caregiver is assessed. This also showed that quantile regression is more precise and powerful than the conventional approach. Moreover, when dealing with continuous predictors, the conventional approach requires categorizing them into intervals; for example, age was categorized into 30–39 years, 40–49 years, and so on. As a result, the reference values for a 40-year-old caregiver are the same as those for a 49-year-old caregiver, but are different from those for a 39-year-old caregiver, even though the difference in age is 9 years in the former case but only 1 year in the latter. This is not reasonable and increases the likelihood of misclassifying caregivers’ QoL status. Instead, the use of age as a continuous predictor in the development of reference values in a regression-based technique is a more principled approach. Compared with other regression-based techniques that mainly aim to estimate the central tendency, quantile regression can explicitly estimate various quantiles (percentiles) conditional on predictor variables. These features are particularly suitable for the construction of reference values. Furthermore, model diagnostic evaluation is important. In this study, we examined the linearity assumption for age and two categorical predictors (caregiver role and performance status), as well as the presence of any significant 2-way interaction effect. We also tested the difference between levels before making a conclusion regarding the suitability of combining groups for categorical predictors. In addition, users of the reference values are reminded that the caregivers in this study were aged between 21 and 79 years, and the findings should not be applied to caregivers of cancer patients outside this age range. Nevertheless, family caregivers of a cancer patient are rarely very young or very old, so the reference values are applicable to the majority of caregivers.

A limitation of the present study is that we collected the data using the full-length SCQOLS and extracted the respective items to calculate the SCQOLS-15 and SCQOLS-10 scores, instead of using a 15-item or 10-item short forms. Hence the interpretation of the findings in this study requires an assumption of no context effects. In other words, the 15 or 10 items selected perform as seen here regardless of whether they are embedded in the full-length form or administered as a standalone short form. However, previous studies have pointed out that there was little context effect in QoL assessment [20–23]. Therefore, we consider this assumption valid. Another limitation is the relatively small number of non-Chinese caregivers. The association between ethnicity and some scores might have been significant, but this study was not sufficiently powerful to detect it. Further studies are warranted.

## Conclusion

We have estimated the percentiles of the SCQOLS-15 and SCQOLS-10 scores. These percentiles can serve as reference values for caregivers in various characteristic-specific subgroups. By comparing their own scores with the reference values, respondents can assess their relative position among the population of family caregivers of cancer patients.

## Supplementary Information

**Additional file 1.**

## Data Availability

The de-identified dataset analyzed is available from ScholarBank@NUS (10.25540/AXN8-5EKD).
